# SBMDb: first whole genome putative microsatellite DNA marker database of sugarbeet for bioenergy and industrial applications

**DOI:** 10.1093/database/bav111

**Published:** 2015-12-07

**Authors:** Mir Asif Iquebal, Sarika Jaiswal, U.B. Angadi, Gaurav Sablok, Vasu Arora, Sunil Kumar, Anil Rai, Dinesh Kumar

**Affiliations:** ^1^Centre for Agricultural Bioinformatics, Indian Agricultural Statistics Research Institute, Library Avenue, PUSA, New Delhi 110012, India,; ^2^Biotechnology Unit, Department of Botany, Jai Narain Vyas University, Jodhpur 342003, India,; ^3^Plant Functional Biology and Climate Change Cluster (C3), University of Technology, Sydney, PO Box 123 Broadway New South Wales 2007, Australia,; ^4^National Bureau of Agriculturally Important Microorganisms, Kusmaur, Mau NathBhanjan, Uttar Pradesh 275101, India and; ^5^Institute of Life Sciences, Nalco Square, Bhubaneswar 751023, India

## Abstract

DNA marker plays important role as valuable tools to increase crop productivity by finding plausible answers to genetic variations and linking the Quantitative Trait Loci (QTL) of beneficial trait. Prior approaches in development of Short Tandem Repeats (STR) markers were time consuming and inefficient. Recent methods invoking the development of STR markers using whole genomic or transcriptomics data has gained wide importance with immense potential in developing breeding and cultivator improvement approaches. Availability of whole genome sequences and *in silico* approaches has revolutionized bulk marker discovery. We report world’s first sugarbeet whole genome marker discovery having 145 K markers along with 5 K functional domain markers unified in common platform using MySQL, Apache and PHP in *SBMDb*. Embedded markers and corresponding location information can be selected for desired chromosome, location/interval and primers can be generated using Primer3 core, integrated at backend. Our analyses revealed abundance of ‘mono’ repeat (76.82%) over ‘di’ repeats (13.68%). Highest density (671.05 markers/Mb) was found in chromosome 1 and lowest density (341.27 markers/Mb) in chromosome 6. Current investigation of sugarbeet genome marker density has direct implications in increasing mapping marker density. This will enable present linkage map having marker distance of ∼2 cM, i.e. from 200 to 2.6 Kb, thus facilitating QTL/gene mapping. We also report e-PCR-based detection of 2027 polymorphic markers in panel of five genotypes. These markers can be used for DUS test of variety identification and MAS/GAS in variety improvement program. The present database presents wide source of potential markers for developing and implementing new approaches for molecular breeding required to accelerate industrious use of this crop, especially for sugar, health care products, medicines and color dye. Identified markers will also help in improvement of bioenergy trait of bioethanol and biogas production along with reaping advantage of crop efficiency in terms of low water and carbon footprint especially in era of climate change.

**Database URL**: http://webapp.cabgrid.res.in/sbmdb/

## Introduction

Sugarbeet (*Beta vulgaris* L. *ssp*. *vulgaris*) is a biennial, dicotyledonous crop of temperate climate. It represents the world’s second highest source of sucrose with 15–20% sugar content (1) after sugarcane (*Saccharum officianarum* L.). It accounts for ∼30% of the world’s annual sugar production and has also been considered as a potential biofuel crop (2) besides its potential as animal feed (3) and medicinal properties (4). With the ever increasing rise in the global population to be around 10 billion in 2050, finding sustainable solutions to the bioenergy research is becoming an important unanswered question. The use of potential food crops for biofuels will be one of the critical needs to support the global projected population. Its increasing importance in bioenergy has led to greater area for production of bioethanol and biogas (5).

Among the largest sugar beet producers, Europe and the United States share 75% of both, global area harvested and production. Among the main producers, France, Germany, the Russian Federation, Turkey and Ukraine, covers almost two thirds of the global production (6). Sugarbeet has been introduced in India in 1971 but its huge industrial potential has not been reaped so far. The demanding biofuel requirement in the country and globe as well, has necessitated the need of ethanol from sugarbeet. Very recently few cases of industrial level production in India, especially from the area of Punjab and Karnataka for sugar and alcohol production, respectively, has been started. If ensilage and anaerobic digestion approach is used, it has further potential of more energy per hectare than bioethanol (7).

Besides industrious use of sugarbeet crop in terms of sugar and bioenergy, it also possesses the additional multifold advantages like: it is tolerant to various climatic and soil conditions thus uncultivable land can also be used. In agriculture, it has three major importance namely, cash crop, soil amelioration/soil fertility improvement and use as by-products for cattle feed/mineral supplement during summer/drought, especially when there is scarcity of green fodder (8).

Beside agricultural importance, sugarbeet plays very important role in industrial area as sunless tanner dihydroxyacetone extracted from sugar beet (9). For human health, it has good medical potentials for anticancerous activity (10) and is a good source of antioxidant (11), aphrodisiac (12), antidepressant (13) and organic dyes (14).

Additionally, it is used in herbal therapy and hepatoprotective activity (4, 15). Furthermore, versatile industrial compounds like betaine (16), phenolics and betacyanins (17) obtained from the sugarbeet are also well documented in literature for their therapeutics. Betain is used in industry for PCR adjuvants as it improves amplification of GC-rich DNA sequences (18). Sugarbeet being, short season crop (6 months), offers advantage over sugarcane (12–18 months) along with its ability as most efficient crop in terms of water foot printing (19) and also for lowering ethanol's carbon footprint (20).

To accelerate the rate of genetic gain for high sugar content, resistance towards biotic (disease causing pathogens) and abiotic stresses (high temperature and saline/alkaline conditions) molecular markers are imperative and have been developed in various crops. Apart from abiotic stresses, sugarbeet is susceptible to over 60 disease caused by pathogens like bacteria, fungi, nematodes, viruses, phytoplasmal, spiroplasmal pathogens, aphids etc. (21–23). Biotic stress can lead to loss even upto 50% of sugarbeet yield (24). Molecular markers play major roles in higher root yield, strong selection against premature bolting, annuality and winter hardness which are the major problems in sugarbeet abiotic management (25).

Present linkage map of sugarbeet constitutes of nine groups with ∼700 cM marker coverage (26, 27). Dohm *et al.* (28) reported an extended genetic map consisting of 983 markers, and Holtgrawe *et al*. (29) in 2014 further added 307 markers to the existing dataset. A sugar beet physical map based on 8361 EST-derived probes was also provided (28). Fugate *et al.* (30) has reported 7680 putative SSR markers.

*In vitro* methods of Short Tandem Repeats (STR) development is disadvantageous as it is time-consuming and expensive. Availability of whole genome sequence and *in silico* approach has revolutionized the marker discovery. Recently, a new class of functionally relevant microsatellites called as simple sequence repeats functional domain markers (SSR-FDMs) (31–33) have gained wide importance. This is being widely applied in a number of crop species including the biofuel and energy crop species such as sugarcane (34). For molecular breeding program of sugarbeet, its recently available genome assembly (569 Mb) of KWS2320 genotype (3) needs *in silico* approach for bulk marker discovery. Further, there is a need of *in silico* discovery of polymorphism of these markers utilizing resequencing data of four additional genotypes namely, KWS230-DH1440, STR06A6001, SynMono and SynTilling. These markers should be in the form of ‘ready to use’ and readily available to the global community in form of freely accessible database.

Our present work aims at development of microsatellite marker database of sugarbeet whole genome-based STR mining. We further aimed, the user defined primer designing with precise selection from each chromosome, at defined location and equal interval along with evaluation of polymorphism. This work also aims at mining of SSR-FDM from various major sources which can be assessed for the genotyping for direct functional markers using genomic DNA primers.

## Material and methods

### Data collection and search flexibilities

For mining of markers, the recently sequenced sugar beet genome data of genotype KWS2320 was used. This haploid line genome was of 567 Mb of which 85% data assigned over its nine chromosomes (2*n* = 18) having an assembly coverage of 63% was used in our study.

This assembly is having more than 27 000 predicted genes (3). This *de novo* assembly was downloaded from http://www.ncbi.nlm.ih.gov/assembly/GCA_000511025.1#/st in FASTA format. These were cleaved using in house PERL scripts and parsed for the identification of the microsatellite markers using the MIcroSAtellite identification (MISA) tool (http://pgrc.ipk-gatersleben.de/misa/) with default parameter setting.

For the mining of the functional SSRs markers (SSR-FDMs), Expressed Sequence Tags (ESTs) were downloaded from NCBI (www.ncbi.nlm.nih.gov). Additionally, Putative Unique Transcripts (PUTs) for suagrbeet were systematically downloaded from PlantGDB (Version release 187) available at http://www.plantgdb.org/. All the ESTs and PUTs were first scanned for the presence of the homopolymers errors and sequence ambiguity was further removed using the est_trimmer available at http://pgrc.ipk-gatersleben.de/misa/download/est_trimmer.pl with the following settings: -amb=2,50 -tr5=T,5,50 -tr3=A,5,50 and were subsequently screened for the SSRs identification using MISA. For the identification of the functional domains, the PUTs were translated into all the coding frames and were searched against Interpro. PUTs having SSRs and Interpro assigned functional domain were classified as SSR-FDMs (31–33, 37). For genotyping of SSR-FDM, primers were designed on genomic DNA sequence.

**Figure 1.**
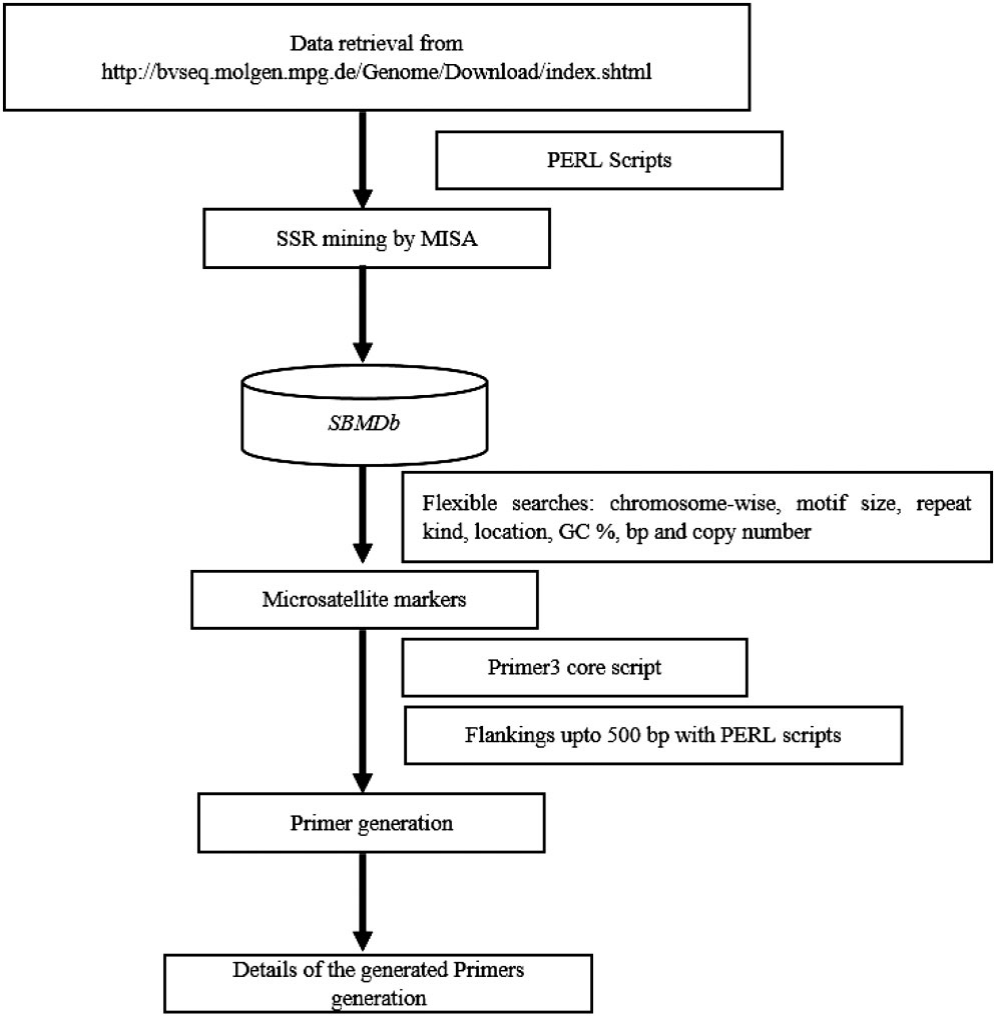
Flow of the database search.

Whole genome based markers were generated with descriptive information on motif size, motif type, repeat numbers with their length and size, repeat type, GC content, start and end position. Provision was made for locating markers on each chromosome at desired interval for mapping of Quantitative Trait Loci (QTL)/gene. Additionally, marker can be selected based on motif type, repeat kind, GC content, number of base pair and copy number of repeat unit as markers with more than eight repeat often exhibits polymorphism due to slippage event in DNA replication. An additional plug-in of primer generation was implemented for the markers, using the primer3 core executable with further flexibility of 500 bp upstream and downstream sequence extraction using PERL scripts targeting approximately 1000 bp as a template for primer designing. Figure 1 demonstrates the flow of analytical pipeline developed for the SBMDb.

For the identified markers, web-based application was created in the window web development environment, WAMP Server with Apache, PHP and MySQL Database.

### Database development

Sugarbeet MicroSatellite Database (*SBMDb)* has been developed using PHP and MySQL database under the web development environment, WAMP Server. This relational database was developed based on ‘three tier architecture’ having client tier, middle tier and database tier. Provision to store all *in silico* mined STRs was made at the backend in MySQL database. PHP scripts were written to properly query and execute the search made by users. The primer3 core was integrated to compute primers of the selected STRs. Primer call for specific locus, i.e. output of primer designing is with list of five primers with their respective melting temperature, GC content, start position and estimated PCR product size are available in the database. Functional domains linked with the simple sequence repeat patterns as an add-on utility to search for the simple sequence repeats functional domain markers (SSR-FDMs) has also been made. To identify the functional domains, all the sequences were translated into all the six reading frames and Interproscan tool was used to analyse and predict the protein domains using the default settings (31–33). Sequences harboring the functional domains and the simple sequence repeats along with the primer pairs were classified as the functional markers. The database has been designed to cater the needs of the plant biologist and breeders thus making it very flexible to access with user defined options. The choice of motif type, namely, mono, di, tri, tetra, penta and hexa, repeat type and repeat kind (simple and composite) over all the nine chromosomes will be useful to breeding researchers and QTL placements to select desired type of STR markers.

### In silico discovery of polymorphic markers

A total of five genotypes namely, KWS2320, KWS230 DH1440 (KDHBv), STR06A6001 (UMSBv), SynMono (YMoBv) and SynTilling (YTiBv) (http://bvseq.molgen.mpg.de/) were used for *in silico* discovery of polymorphic markers using selected SSRs. Since polymorphism is exhibited by SSR having greater than or equal to eight repeat unit (35), these were selected and all simple repeats except ‘mono-nucleotide’ repeats were selected for discovery of polymorphic markers. For this, in house perl scripts were written accordingly. Further, selected primers were put in e-PCR (36) among five genotypes. Locus having difference in PCR product size were considered as polymorphic.

## Results and discussion

### Analysis of sugarbeet genome and relative abundance

**Figure 2.**
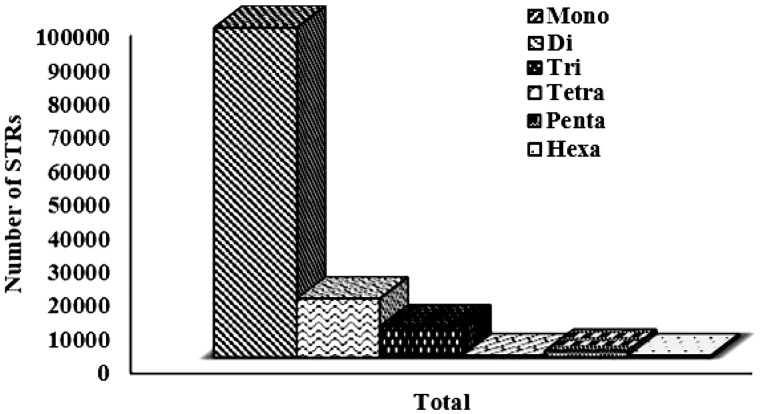
Graphical representation of motif-wise distribution of microsatellites in sugarbeet genome.

The overall analysis of available sugarbeet genome gives the association of the distribution of the microsatellite markers to the genomic attributes. A total of 145 K STR markers were successfully mined and populated in database as user friendly application. The distribution of simple and compound repeat types were 88 and 12%, respectively. Among simple type, ‘mono’ repeat type were more prevalent with 76.82%, followed by ‘di’ repeats, which was 13.68%. Although ‘di-nucleotide’ repeat type are observed abundantly in eukaryotes (38), on the contrast, our analysis reports ‘mono’ repeat patterns as the most abundant type (Figure 2). Since MISA parameters were not set for any threshold for mono-repeats, thus this prominence might be due to the inherent limitation of the NGS technology used which causes more mono nucleotide stretches as sequencing error (39).

**Figure 3.**
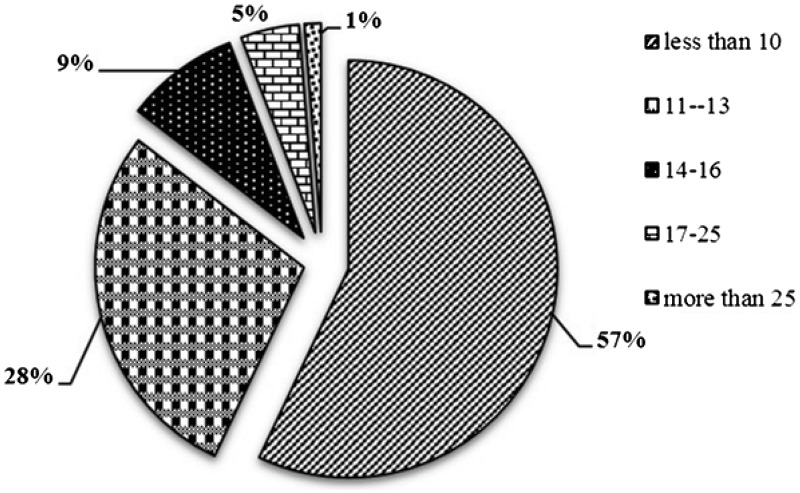
Distribution of microsatellite sizes in sugarbeet genome.

STR markers being ubiquitously distributed, proportionately higher repeat content for longer chromosomes are expected (40), which is also observed in the present analysis. The most abundance STRs were distributed in Chromosome 1, followed by Chromosome 6 and 5, while Chromosome 3 contains the least abundant STRs (Table 1). The proportion of STRs with size less than (<10 bp) was maximum (57.05 %) followed by the ones between the size range of 11–13 bp (28.38%) and size range 14–25 bp (13.32%). Only 1.26% of the total STRs belonged to the size more than 25 bp (Figure 3). Chromosome 1 showed highest density (671.05 markers/Mb) of markers and chromosome 6 reports minimum density of markers (341.27 markers/Mb), while the relative density of the sugarbeet whole genome is 378.54 markers/Mb, showing that these markers are ubiquitously distributed with homogeneity in terms of distance, which is inherent attribute of microsatellite to be used as marker of choice. Remaining all seven chromosomes were having the marker density of 341.27 to 384.94 marker/Mb.

**Table 1.** Motif-wise distribution of microsatellites in sugarbeet genome

Chromosome	Simple						Simple	Compound
	Mono	Di	Tri	Tetra	Penta	Hexa		
1	16 014	2896	1502	76	284	80	20 852	2930
2	9970	1675	947	43	212	46	12 893	1737
3	7200	1089	643	33	159	41	9165	1212
4	8302	1277	754	41	179	46	10 599	1470
5	12 125	2325	1187	70	235	67	16 009	2189
6	14 132	2624	1336	83	265	70	18 510	2592
7	10 158	1895	917	47	201	61	13 279	1758
8	9035	1704	830	54	170	57	11 850	1688
9	10 723	1906	1012	55	217	61	13 974	1882
Total	97 659	17 391	9128	502	1922	529	127 131	17 458

The relative density of the sugarbeet whole genome reported in the present study is 379 markers per Mb, which is more than the range in Arabidopsis (157 markers per Mb). The other crops having similar number of markers are, cucumber (367 markers per Mb), rice (370–490 markers per Mb), poplar (485 markers per Mb) and grape (487 markers per Mb).

The initial linkage map of sugarbeet was having nine groups, with 700 cM coverage with just 500 STR markers (26, 27). An extended genetic map of sugar beet (*Beta vulgaris* L.) was achieved with 177 segregating markers on nine linkage groups (26). The linkage map comprises 1057.3 cM. Marker density calculations of present genetic map reveal a distance of ∼2 cM between markers. The bulk set of markers (145 K), identified in the present study were assigned to the projected physical map and showed 430-fold higher marker density i.e. segregating two markers with a distance of 2.6 Kb. Since the average size of any eukaryotic gene falls within this distance between markers. Thus, these set of markers can ensure mapping of almost all genes.

In evaluation of 15 513 repeats by e-PCR, we found 2027 polymorphic markers in panel of five genotypes. Chromosome-wise distribution is summarized in Table 2 and details are given in supplementary table (Supplementary Table 1).

**Table 2.** Chromosome-wise number of polymorphic markers

	Difference in product size between reference genotype and				
Chromosome	All 4	At least 3	At least 2	At least 1	Total
1	4	18	37	151	210
2	5	9	35	158	207
3	3	3	28	121	155
4	2	7	36	129	174
5	2	14	39	229	284
6	1	12	64	260	337
7	6	12	33	188	239
8	1	11	48	138	198
9	2	13	40	168	223
Total	26	99	360	1542	2027

## Utility of the database

Previously, several attempts have been made for increasing the markers based species delineation and genus identification events in *Beta vulgaris*. Earlier attempts have been made using the morphological descriptor and isozyme markers to differentiate *Beta vulgaris* and *B webbiana* (41). Earlier attempts have been made to delineate the approaches for the varieties/lines differentiation within the *B. vulgaris* species using both STR and SNP markers.

Varieties/line differentiation within the species of *B. vulgaris* has been attempted by both STR and SNP markers, e.g. a limited 677 SNP markers have been used for differentiation of 924 lines of sugarbeet (42).

However, there is limited use of STR markers in sugarbeet variety identification as reported earlier (43). Additionally, the number of informative morphological characters is limited in sugarbeet that often leads to some problems in variety registration (43). Previously there have been reports on the varietal differentiation using 12 STR markers in this species (43). However, the amount of the markers used were very few, which is a bottle-neck in this species. In the present report, the identified 145 K markers, can serve as a good reference resource for the development of the varietal identification markers. These whole genome markers have also played a role in the mapping and variety identification supplementing Distinctness, Uniformity and Stability (DUS) test and product trace ability (44). Use of STR in plant variety identification is well reported in other crops like barley varieties (45), S*. tuberosum ssp. tuberosum* (46), sugarcane (47), capsicum (48) and identification of Basmati rice from that of non-Basmati rice (49) etc.

STR markers have known to play an important role in regulating the gene expression. The observed markers in the present study are ubiquitously distributed can help in deciphering the gene level regulation. For example, length changes of microsatellites within promoters and other *cis*-regulatory regions can also change gene expression quickly, between generations. Such mechanism is already reported in large number of genomes. For example, in case of human genome, more than 16 000 STRs in regulatory regions are working as ‘tuning knobs’ for gene expression (50). Additionally, STR markers distributed across the intronic regions are reported to influence phenotype (51, 52). Such relation of STR with phenotype has not been reported in *Beta vulgaris*. Our markers can be used for exploration of similar association. Transposable element contributes in plant gene regulation (53). Such transposable elements are present in sugarbeet also (54). It is probable that short sequence repeats in those locations are also involved in the regulation of gene expression (55). The repeat sequence mined in our database can be used for such studies where transposable elements play role in gene regulation.

STR has been used to trace hybridization and introgression events with wild beet to monitor feral or wild beet characters in GM beets (56), genetic diversity and root traits (57). Similarly, SNP markers have also been used for diversity analysis of sugarbeet (57). Our *in silico* discovered 2027 polymorphic markers can also be used for diversity studies and phylogenetic studies of varieties or species.

A deep review of the previously published literature illustrates that identification of candidate genes for marker-assisted selection can improve the efficiency of breeding for increased drought tolerance (58). Need of markers to improve crop efficiency ratio in alkali soil for alcohol production is reported by Garg and Khanduja (59). Molecular markers are needed for mapping of disease resistance genes by linkage analysis in sugarbeet (60). Use of molecular markers are reported for construction of linkage map and identification of commercially valuable CMS in sugarbeet (61). Genes for various economically and commercially relevant trait of sugarbeet has been reported, e.g. seedling vigour (62), FLC-like gene BvFL1 associated with annuality and winter hardiness (25), root traits (57), non-restoring allele for Owen-type cytoplasmic male sterility, for development of molecular markers for the maintainer genotype (61), aphid resistance (63), nematode resistance (64), QTL for leaf spot (65), hardiness and bolting (66) and draught and salt tolerance (67). To increase the crop efficiency for bioenergy mapping of bioenergy traits are imperative (68). Markers can be used as a genomic resources to increase the biofuel potential from sugarbeet (30). We believe that the genome-wide STR makers developed and displayed graphically in our database with the options to synthesize the primers directly for the desired regions of the chromosome will serve the ease of developing markers for screening the mapping-based population for the genes involved in the several key domestication and biofuel traits.

Additionally, *SBMDb* provides access to the first ever comprehensive catalogue of the SSR-FDMs along with the markers from the genome wide coverage. Previously, SSR-FDMs have been widely used for the fluorescent based markers with an average of 7.42 alleles per locus in sugarcane (34). Utility of these markers also established the structure–function relationship for the beta-amylase and protein kinase encoding unigenes, which harbors the functional repeats in the catalytic domains (34). It is worthwhile to mention that a high robust amplification efficiency (96.5%) and high intra-specific polymorphic potential (34%) has been recently been exploited for the genotyping and trait association mapping in Chickpea using the 1108 transcription factor gene-derived microsatellite (TFGMS) and 161 transcription factor functional domain-associated microsatellite (TFFDMS) markers (69). Based on the above observations and later on application of the SSR-FDMs in several crop species such as *Ocimum basilicum* (70), *Seasme indicum* (71), *Elaeis guineensis* (72) and *Camellia sinensis* (73), suggests that linking the identified markers to the possible functional domains extends the evaluation of these markers from genotypic arrays and possibly can help us to elucidate the possible linkage of the strand slippage mechanism to the functional relevance.

If STR markers from our database are used for mapping of these genes or markers of flanking regions of these specific genes are selected, then they can directly be used in molecular breeding program for introgression of these genes/traits.

## Conclusion

Using a computationally intensive *in silico* approaches, we mined and catalog the 145 K STR markers and built the first whole genome based STR database, which is freely accessible to the public domain at http://webapp.cabgrid.res.in/sbmdb/. With the marker information present in the *SBMDb*, the linkage map’s marker density can be increased which will facilitate in QTL and gene mapping. In order to facilitate the use of these markers in various molecular breeding and QTL programs, we have implemented several plug-in to generate primers at user defined chromosomal locations, which can be directly exported for genotyping assays. Additionally, the identified polymorphic markers can also be used for the DUS test for variety identification and improvement, MAS/GAS, QTL and gene mapping and germplasm improvement and management through marker genotyping. The present database will overcome the need of the marker portal of the sugarbeet genomics and the user friendly design will also help in the easy to access the marker information for molecular breeding required to accelerate industrious use of this crop, especially for sugar, biofuel/bioenergy, health care products, medicines, color dye. These markers need widest utilization across globe for best industrious use of sugarbeet by improvement of bioenergy trait of bioethanol and biogas production. This will not only improve the crop efficiency, but will also be a model industrial crop in the endeavour of water and carbon footprint in the challenging climate change regime.

## Availability and requirement

*SBMDb,* the sugarbeet microsatellite marker database is freely accessible for research purposes for non-profit and academic organizations at http://webapp.cabgrid.res.in/sbmdb/.

## Supplementary Data

Supplementary data are available at *Database* Online.

Supplementary Data
