# Supplementary material for: SBMDb: first whole genome putative microsatellite DNA marker database of sugarbeet for bioenergy and industrial applications
Source: Database (Oxford). 2015 Dec 7;2015:bav111. doi: 10.1093/database/bav111 (PMC4672366; doi:10.1093/database/bav111)
Supplement: Supplementary Data [file supp_2015_bav111_index.html]

SBMDb: first whole genome putative microsatellite DNA marker database of sugarbeet for bioenergy and industrial applications — Supplementary Data 

# SBMDb: first whole genome putative microsatellite DNA marker database of sugarbeet for bioenergy and industrial applications

## Supplementary Data

files

- Supplementary Data - xlsx file
- Supplementary Data - doc file
