# Supplementary material for: SBMDb: first whole genome putative microsatellite DNA marker database of sugarbeet for bioenergy and industrial applications
Source: Database (Oxford). 2015 Dec 7;2015:bav111. doi: 10.1093/database/bav111 (PMC4672366; doi:10.1093/database/bav111)
Supplement: Supplementary Data [file supp_bav111_Supplementary_Table_1.doc]

**Supplementary Table 1**. Chromosome-wise list of polymorphic markers along with the primer information and product size.
